# Supplementary material for: A Posteriori Dietary Patterns and Coronary Artery Disease in a Greek Case–Control Study
Source: Nutrients. 2023 Nov 9;15(22):4733. doi: 10.3390/nu15224733 (PMC10675662; doi:10.3390/nu15224733)
Supplement: Supplementary file 1 [file nutrients-15-04733-s001.zip › nutrients-2653732-supplementary.pdf]

Supplementary material

Table S1. Assessment of food groups

Refined starch	Refined breakfast cereals, chocolate cereals, white toast bread, white bread, sesame bagel, rice, spaghetti,
Non-refined starch	All bran cereals, muesli, whole wheat toast bread, whole wheat bread
Fruits	Apple, orange, banana, pear, kiwi, tangerine, apricots, peach, watermelon, melon, strawberries, figs, grapes, cherries, dried figs
Fresh fruit juice	Fresh fruit juice, fruit juice (100%)
Fruit juice	Fruit juice (<100%)
Vegetables	Tomatoes, cucumber, onions, turnip, garlic, carrot, lettuce, cabbage, spinach, broccoli, greens, eggplants, pumpkins, artichokes
Potatoes	Baked potatoes, boiled potatoes, smashed potatoes
Potatoes	Fried potatoes
Legumes	Lentils, beans
Fish (including fried fish)	small fish, big fish
Poultry	Chicken breast, chicken leg
Red meat	Pork, beef, lamb/goat, liver
Processed meat	Salami, sausage, turkey, bacon,
Dairy, full-fat	Milk full fat, yogurt full fat
Dairy, semi-fat	Milk, semi-fat, yogurt semi-fat
Dairy, non-fat	Milk non-fat, yogurt semi-fat
Cheese, full-fat	Feta, parmesan, gruyere
Cheese, semi-fat	composite cheese
Eggs	Egg boiled, egg fried/scrambled
Fast foods	Pizza, cheese pie, ham-cheese pie, spinach pie
Sweets	Chocolate almond, dark chocolate, mini chocolate, chocolate croissant, cookies, chocolate cake, ice cream, ravani, baklava, pecan pie, donut, nougat, halvah, sugared bun, melomakarono
Nuts and seeds	Almonds, peanuts, hazelnuts, cashew, walnuts, pine nut
Beverages with sugar	Soft drinks
Beverages, non-sugar	Soft drinks light
Beverages with alcohol	Beer, red wine, white wine, whiskey, ouzo, liquor,
Coffee	Coffee
